# Fe(III) bioreduction kinetics in anaerobic batch and continuous stirred tank reactors with acidophilic bacteria relevant for bioleaching of limonitic laterites

**DOI:** 10.3389/fmicb.2024.1358788

**Published:** 2024-03-11

**Authors:** Agathe Hubau, Catherine Joulian, Hafida Tris, Douglas Pino-Herrera, Camille Becquet, Anne-Gwénaëlle Guezennec

**Affiliations:** BRGM, Orléans, France

**Keywords:** reductive bioleaching, iron bioreduction, sulfur biooxidation, ferric iron, acidophilic microorganisms, anaerobic

## Abstract

In the framework of the H2020 project CROCODILE, the recovery of Co from oxidized ores by reductive bioleaching has been studied. The objective was to reduce Fe(III) to Fe(II) to enhance the dissolution of Co from New-Caledonian limonitic laterites, mainly composed of goethite and Mn oxides. This study focused on the Fe(III) bioreduction which is a relevant reaction of this process. In the first step, biomass growth was sustained by aerobic bio-oxidation of elemental sulfur. In the second step, the biomass anaerobically reduced Fe(III) to Fe(II). The last step, which is not in the scope of this study, was the reduction of limonites and the dissolution of metals. This study aimed at assessing the Fe(III) bioreduction rate at 35°C with a microbial consortium composed predominantly of *Sulfobacillus* (*Sb.*) species as the iron reducers and *Acidithiobacillus* (*At.*) *caldus*. It evaluated the influence of the biomass concentration on the Fe(III) bioreduction rate and yield, both in batch and continuous mode. The influence of the composition of the growth medium on the bioreduction rate was assessed in continuous mode. A mean Fe(III) bioreduction rate of 1.7 mg·L^−1^·h^−1^ was measured in batch mode, i.e., 13 times faster than the abiotic control (0.13 mg·L^−1^·h^−1^). An increase in biomass concentrations in the liquid phase from 4 × 10^8^ cells·mL^−1^ to 3 × 10^9^ cells·mL^−1^ resulted in an increase of the mean Fe(III) bioreduction rate from 1.7 to 10 mg·L^−1^·h^−1^. A test in continuous stirred tank reactors at 35°C resulted in further optimization of the Fe(III) bioreduction rate which reached 20 mg·L^−1^·h^−1^. A large excess of nutrients enables to obtain higher kinetics. The determination of this kinetics is essential for the design of a reductive bioleaching process.

## 1 Introduction

Cobalt (Co) has exceptional high-temperature strength, corrosion resistance, catalytic, and electrochemical properties, which make it a crucial element for high-performance applications. Due to the development of these applications, and in particular lithium-ion batteries, the cobalt demand is growing rapidly. Co was thus classified by the European Commission as a critical raw material (European Commission, 2020). In this context, the CROCODILE project aimed at developing innovative metallurgical systems based on advanced pyro-, hydro-, bio-, iono-, and electrometallurgy technologies for the recovery of Co and the production of Co metal and upstream products from a wide variety of secondary and primary European resources (Crocodile Project, 2018).

During primary mining, Co is with few exceptions a by-product of copper and nickel (Ni) primary production. The majority of global Ni resources are hosted by laterite deposits and the remaining portion in sulfide deposits (Mudd and Jowitt, 2014). Owing to the exhaustion of higher grade sulfidic deposits as well as to the increase in demand for Ni, the production of Ni from laterites continues to increase. Laterite deposits are characterized by the presence of two distinct zones:

- A limonite zone near the surface where Ni is mainly hosted in ferric iron oxides, typically goethite (FeOOH) and to a lesser extent in Mn oxides. Ni content usually varies between 1 and 2%.
- A lower saprolite zone that is dominated by hydrous magnesium silicates (MgO > 20%), with a nickel content varying between 1.5 and 2.5%.

Saprolite is usually treated via pyrometallurgical reduction. In contrast, limonite cannot be economically treated via pyrometallurgy due to the combination of a high Fe and a low Ni content. Limonite ore is traditionally processed through pressure acid leaching (PAL). Unfortunately, PAL plants have often proven to be very costly, plagued with technical challenges (Oxley et al., 2016). Still today, a large proportion of limonite ore is stockpiled for future processing. Reductive bioleaching is one of the metallurgical options that are currently studied to extract Co and Ni from limonite.

During reductive bioleaching, the main following reactions occur:


(1) $$FeOOH+3H^{+}↔Fe^{3+}+2H_{2}O$$

(2) $$6Fe^{3+}+S^{0}+4H_{2}O\overset{bacteria}{\to }6Fe^{2+}+SO_{4}^{2-}+8H^{+}$$

(3) $$MnO_{2}+2Fe^{2+}+4H^{+}\to Mn^{2+}+2Fe^{3+}+2H_{2}O$$

Goethite equilibrium in the acid environment is shifted toward its dissolution (Eq. 1), due to acid generation and bioreduction of Fe(III) to Fe(II) (Eq. 2). This bioreduction also produces the ferrous iron involved in Mn oxide dissolution (Eq. 3). The concept of reductive bioleaching was first introduced by Hallberg et al. (2011). Since then, several studies dealing with the proof of concept of reductive bioleaching have been released (Johnson and du Plessis, 2015; Marrero et al., 2015; Santos et al., 2020, 2022; Johnson and Pakostova, 2021; Stankovic et al., 2022). These studies are mainly dedicated to the study of microorganisms, with a microbiological approach. Very few studies are dealing with process engineering approaches (kinetics data, mass transfer phenomena, etc.). In anaerobic conditions, existing studies only report bioreduction kinetics data obtained with *Acidithiobacillus (At.) ferrooxidans*. In particular, Kucera et al. (2012) revealed a bioreduction rate of 26.1 mg·L^−1^·h^−1^ while in the study of Pronk et al. (1992) bioreduction rate reached 25.6 mg·L^−1^·h^−1^. A recent study from Breuker and Schippers (2024) reported a value of 19.9 mg·L^−1^·h^−1^ in anaerobic conditions in the presence of 10^7^ cells·mL^−1^ for *At. ferrooxidans* after tetrathionate growth in aerobic conditions. However, it is admitted that the use of microbial consortia generally increases the kinetics compared to pure cultures, due to the synergistic effects between microorganisms (Mahmoud et al., 2017). Thus, determining bioreduction kinetics in the presence of microbial consortia is of great interest. Obtaining kinetics data (and in particular Fe(III) bioreduction kinetics as one of the key reactions for the process) is necessary for reactor sizing and other engineering aspects, which will impact the economic and environmental assessments of such processes.

The objective of this study was thus to determine Fe(III) bioreduction kinetics in anaerobic conditions (Eq. 2) in the presence of a microbial consortium containing *Acidithiobacillus* and *Sulfobacillus* species. Due to the limited growth of microbial communities under anaerobic conditions, microbial growth was first performed in aerobic conditions using sulfur as an electron donor, followed by Fe(III) bioreduction kinetics experiments. The microbial consortium was initially composed of different microorganisms and was grown at 35°C. The ferric iron sulfate (Fe_2_(SO_4_)_3_) bioreduction rate was first measured in batch mode. It was then determined in the presence of different biomass concentrations in the liquid phase (10^8^ cells·mL^−1^ and 10^9^ cells·mL^−1^). Finally, the Fe(III) bioreduction rate was evaluated in continuous mode in the presence of two different growth media.

## 2 Materials and methods

### 2.1 Growth media

Two different growth media were used. One, named “ABS-TE medium”, contained acidophilic basal salts (ABS) and trace elements (TE) (Ñancucheo et al., 2016) supplemented with 100 μmol·L^−1^ ferrous iron sulfate and pH adjusted to 2.0 with sulfuric acid. The second one was the so-called 0Km medium (Collinet-Latil, 1989), which is much more concentrated in nutrients than the ABS-TE medium but does not contain any iron.

In some experiments, the medium was supplemented with Fe(II) or Fe(III), through the addition of FeSO_4_.7H_2_O (Acros Organics) or Fe_2_(SO_4_)_3_.xH_2_O (Alfa Aesar). Two types of sulfur were used: (i) laboratory-grade sulfur (sulfur powder −100 mesh refined from Aldrich); (ii) coarse sulfur (sulfur pearls from Aquamedic that were slightly milled with bar crusher and sieved to keep the particles between 400 μm and 2.5 mm).

### 2.2 Culture

The microbial consortium that was used in this study was provided by Prof. Barrie Johnson from the Bangor Acidophile Research Team at Bangor University, United Kingdom. Upon reception, the consortium was subcultured in ABS medium (Ñancucheo et al., 2016) supplemented with 0.5% (w/v) elemental sulfur in shake flasks. The culture was incubated at 35°C for 4 days on a shaker at 100 rpm, and microbial growth was checked via pH measurements and cell counting (see Section 2.5). Fresh medium was inoculated again, and after several times of subculturing, the microbial consortium composition was checked via tRFLP (described in Section 2.5). *Acidithiobacillus (At.) caldus* was identified as the dominant species in addition to *Sulfobacillus (Sb.) thermosulfidooxidans* (relative proportion of 24%).

### 2.3 Bioreduction in batch mode

#### 2.3.1 Stirred tank reactor (STR) equipment

Bioreduction experiments were performed in batch mode in laboratory-scale glass bioreactors with a working capacity of 2 or 4 L. The bioreactors were jacketed for warm water circulation to maintain a constant operating temperature. The temperature of the circulating water was controlled by a cryothermostat and thermocouples placed in the bioreactors. The bioreactors were equipped with four baffles mounted 90° apart and extended down to the base of the vessel to optimize the mixing of pulp. The agitation was performed using a dual impeller system (axial/radial) consisting of a standard 6-blade Rushton turbine combined with a 3-blade 45° axial flow impeller. The gas supply system was designed to accommodate air or N_2_, enriched or not with 1% CO_2_ (v/v). The gas was injected beneath the turbine at the bottom of the bioreactors via a stainless steel pipe. The impellers and the gas injection pipe were positioned to respect the standard dimensions and thus to optimize gas mass transfer and mixing in the bioreactors. The top of the reactors was connected to a gas cooling system to prevent excessive evaporation.

#### 2.3.2 Operating conditions

**Figure 1:**
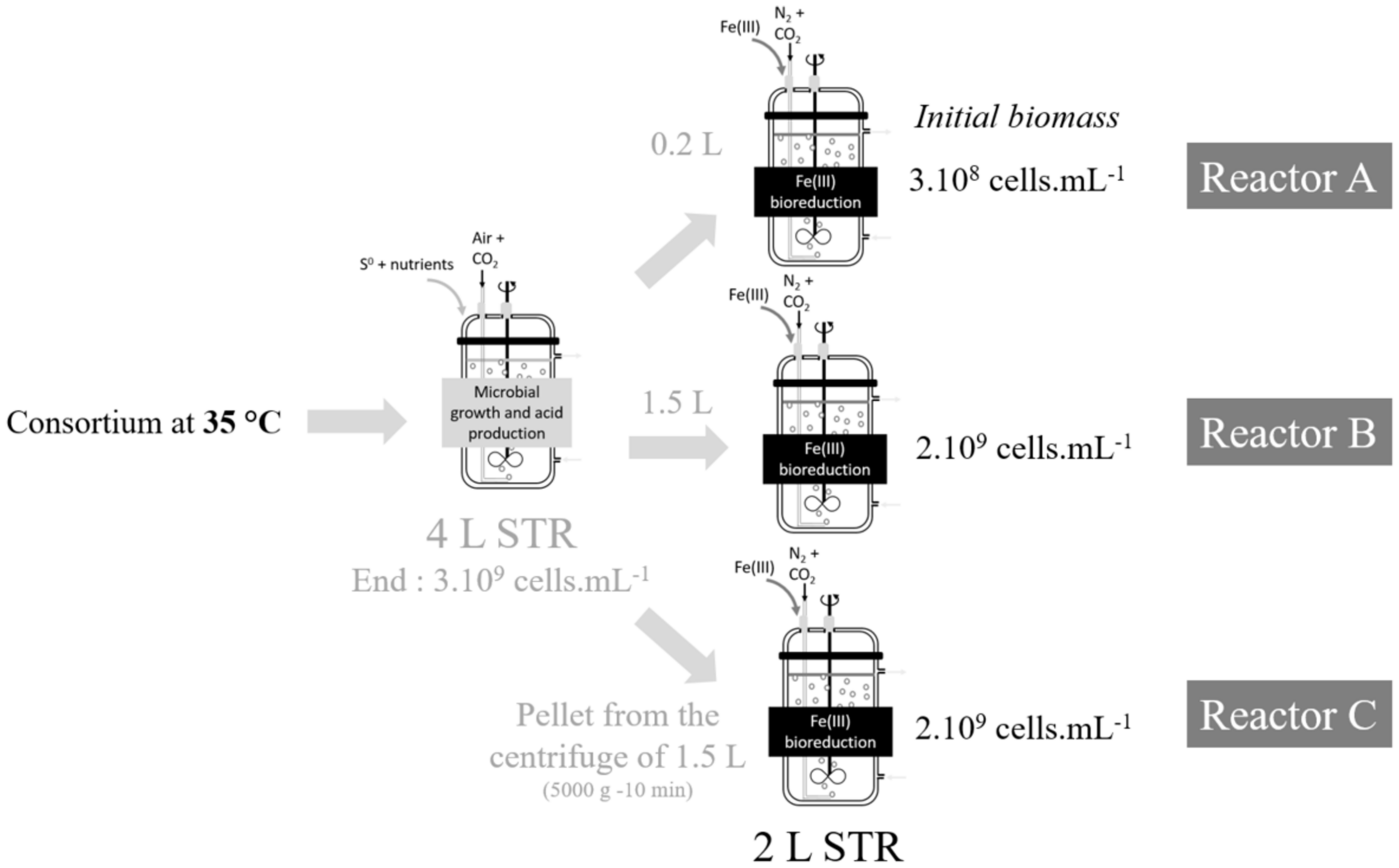
Schematic diagram of the experiments performed in batch mode at different initial biomass concentration at 35°C.

The growth phase was performed in aerobic mode on elemental sulfur in a 4 L-STR (Figure 1). Bioreactor was inoculated with 10% (v/v) in the ABS-TE medium supplemented with 1% (w/w) laboratory-grade sulfur, and the air flow rate was 425 nmL·min^−1^ with 1% CO_2_ (v/v). At the end of the growth phase, pulp biomass concentration reached 3 × 10^9^ cells·mL^−1^ (see Section 2.5 for the methodology for cell counting). *At. caldus* was dominant over *Sulfobacillus* and represented about two-third of the pulp bacteria (see Section 2.5 for the methodology for microbial community monitoring). This pulp was then used to inoculate three bioreduction STRs (2 L) containing 4.5 g·L^−1^ Fe(III). In reactor A, inoculation was done with 200 mL (10% (v/v), corresponding to an initial biomass concentration in the 2 L-STR of 3 × 10^8^ cells·mL^−1^). In reactor B, inoculation was performed with 1,500 mL (75% (v/v), corresponding to an initial biomass concentration in the 2 L-STR of 2 × 10^9^ cells·mL^−1^). In reactor C, inoculation was performed with a pellet obtained after centrifugation of 1,500 mL of pulp at 5000 g for 10 min (corresponding to an initial biomass concentration in the 2 L-STR of 2 × 10^9^ cells·mL^−1^). All bioreduction reactors were gassed with N_2_ (425 nmL·min^−1^) supplemented with 1% CO_2_ (v/v), and the stirring speed was set to 500 rpm. Dissolved oxygen was punctually measured with a FDO 925 probe (from WTW) to ensure that anaerobic conditions were maintained.

### 2.4 Bioreduction in continuous mode

**Figure 2:**
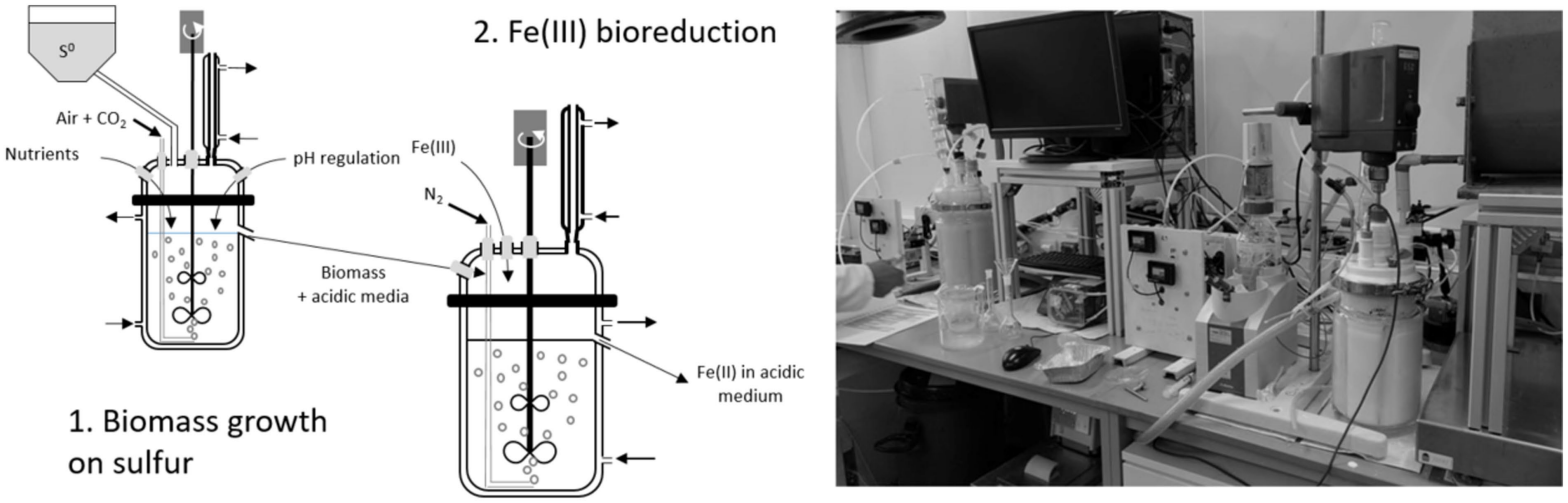
Schematic diagram and image of the experimental device used during the operation in continuous mode.

The experimental devices used in batch mode were also used in continuous mode. The operation was performed in a cascade pilot, with a 2 L-STR in the first step (biomass growth) called “R1” and a 4 L-STR in the second step (Fe(III) bioreduction) called “R2” (Figure 2).

The pilot experiment was started by inoculating the 2 L-STR (R1) in batch mode. When the pH reached 1.5, the continuous mode was started. The pilot was operated in two stages, corresponding to two different growth media. During the first stage, the medium was ABS-TE, while in the second stage, it was 0Km medium supplemented with 1.5 g·L^−1^ Fe(II) (Table 1). In both cases, the pH of the medium was set to 2.0, and coarse elemental sulfur (2.4% w/v) was used.

**Table 1:** Measured growth media composition (in mg·L^−1^) during the different stages of the bioreduction continuous operation.

	Stage 1	Stage 2
NH_4_^+^	127	979
K^+^	70	273
Fe(II)	6	1,468
SO_4_^2−^	1,520	6,402
PO_4_^3−^	36	435

A peristaltic pump was used to add the medium in continuous mode in the first STR (R1). The incoming flow rate was set at 26.5 mL·h^−1^ so that the hydraulic residence time (HRT) was 70 h. Small deviations happened, thus leading to a HRT in R1 of 73 h (± 2 h) during the first stage and 63 h during the second stage. The air flow rate in R1 was set to 885 nmL·min^−1^ enriched with 1% CO_2_. The STR was also fed with coarse sulfur by means of a dosing screw (Dosapro Milton Roy) controlled with a programmable logic controller (Siemens LOGO!). The flow rate of solids was set to 0.675 g·h^−1^ so that the solid concentration was 2.4% (w/v). To adjust the pH to 1.5 in R1, K_2_CO_3_ at a concentration of 4 mol·L^−1^ was added with a peristaltic pump. STR stirring speed was set at 650 rpm, and the temperature was maintained at 35°C.

The outflow (pulp) from R1 was removed with a peristaltic pump and injected into the Fe(III) bioreduction reactor (R2) that was fed continuously. The theoretical HRT was 128 h in the R2, with a stirring speed of 570 rpm. As for R1, similar deviations were observed on R2 HRT, which reached 135 h during stage 1 (ABS-TE medium) and 111 h during stage 2 (0Km medium). A peristaltic pump was used to inject a Fe(III)-rich solution (approximately 22 g·L^−1^) in this reactor with a flow rate of 3 mL·h^−1^ (total Fe concentration in the reactor was approximately 2.4 g·L^−1^). N_2_ was continuously injected to obtain anaerobic conditions (150 nmL·min^−1^). Dissolved oxygen was punctually measured with an FDO 925 probe (from WTW) to ensure that anaerobic conditions were maintained.

### 2.5 Monitoring of the bioreduction experiments

The pH value and the redox potential were measured daily for all experiments. When the pH was controlled at a fix value, H_2_SO_4_ 96% or K_2_CO_3_ 2 or 4 mol·L^−1^ was added manually to reach the set point. Water was added on a daily basis (visual determination of the level of the pulp in the reactor) to compensate water losses due to evaporation. The correlation from Yue et al. (2014) was used to determine the Fe(III) to Fe(II) concentration ratio from the redox potential measurement. The estimation of the bioreduction rate was based on Fe(II) concentration estimation because Fe(III) tends to precipitate by contrast to Fe(II). In batch mode, two rates were determined: (i) The maximal bioreduction rate was determined through the maximal increase of Fe(II) concentration between two measures; (ii) the mean bioreduction rate was determined through the Fe(II) production rate between the beginning and the end of the bioreduction period.

Concentrations of Fe and K in the liquid phase were determined by microwave plasma atomic emission spectroscopy (MP-AES) using a MP-AES 4210 from Agilent Technologies. Mg concentrations were determined by inductive coupled plasma-optical emission spectrometry (ICP-OES, Horiba Jobin Yvon Ultima 2). The concentration of SO_4_^2−^ was determined by ion chromatography (with 940 professional IC Vario from Metrohm). NH_4_^+^ and PO_4_^3−^ concentrations were determined by colorimetric assays in accordance with the NF ISO 15923-1 standard.

The number of microorganisms in the liquid phase was monitored by microscopic counting with a Thoma cell counting chamber. For DNA-based analyses, pulp samples (2 mL) were centrifuged and the pellets were washed by re-suspension in Tris buffer (100 mM, pH 8) until nearly pH 7. The microbial DNA was extracted from the washed pellets with the FastDNA Spin Kit for Soil and using the manufacturer’s protocol (MP Biomedicals) and a FastPrep^®^-24 instrument at a speed of 5 m·s^−1^ for 30 s. The bacterial community structure was assessed by molecular fingerprinting of the 16S rRNA gene as a universal marker of bacteria. Community fingerprints were obtained by tRFLP, as described in Hedrich et al. (2016). The software BioNumerics (Applied Maths) was used to realign the peak profiles based on internal standard migration, assign peak positions, and calculate relative abundances (proportion) on the basis of peak heights. The comparison of the peaks’ position with the peak profile of reference bioleaching strains allowed to distinguish *Sulfobacillus* species (*thermosulfidooxidans*, *thermotolerans*, *acidophilus*) from *Acidithiobacillus* species, *Sb. acidophilus* from *Sb. thermosulfidooxidans*/*thermotolerans*, and *At. caldus* from other *Acidithiobacillus* species (*ferridurans*, *ferrooxidans*, *ferrivorum*).

In the case of the continuous experiment, the taxonomic composition of the microbial community members was also determined, using metabarcoding of the V3–V4 16S rRNA gene region targeted with primers 341F (5′-CCTAYGGGRBGCASCAG-3′) and 806R (5′-GGACTACNVGGGTWTCTAAT-3′). Amplicon libraries, Illumina MiSeq sequencing, base calling, demultiplexing, and Fastq generation were performed by the ADNid sequencing company (Montpellier, France). The bioinformatics pipeline FROGS (Escudié et al., 2017) was used, as described in Joulian et al. (2020). After taxonomic affiliation using the 138.1 Silva database, filtration was done at a minimum identity of 97% and minimum coverage of 99%. Normalization was performed by random resampling of the sequences to have an equal number of 6,081 good-quality sequences for each sample. Operating taxon units (OTUs) retrieved in the PCR control were considered as contaminant sequences and therefore removed. Finally, seed sequences of OTU were subjected to comparison with GenBank 16S rRNA gene reference sequences using Blastn and the implemented distance tree of result tool,^1^ to refine OTU taxonomy, when affiliated to a same genus or family. After all these steps, a clean OTU table was generated. Raw Fastq data are available under the BioProject PRJEB71394.

## 3 Results

### 3.1 Kinetics of ferric iron sulfate bioreduction in batch mode

Kinetics of Fe(III) bioreduction in anaerobic conditions were first evaluated in batch mode. The influence of the initial biomass concentration on the bioreduction rate was determined. Biomass was grown on sulfur under aerobic conditions. Different amounts of inoculum were then added to STR used to perform the bioreduction.

**Figure 3:**
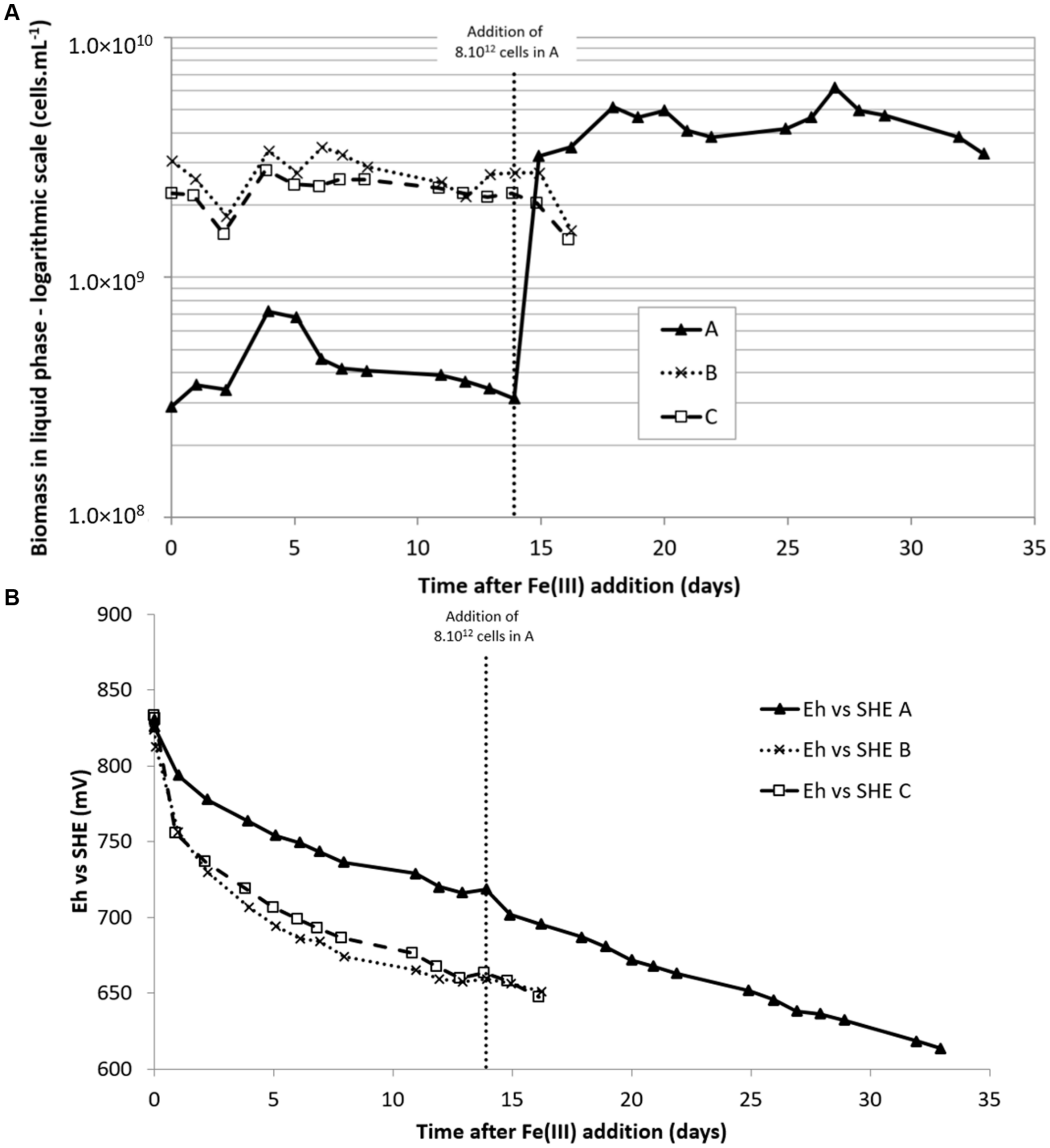
Biomass concentration in the liquid phase **(A)** and redox potential **(B)** during Fe(III) bioreduction experiments at 35°C. Conditions A and B: inoculations were done with 200 mL and 1,500 mL, respectively, corresponding to an initial biomass concentration in the 2 L-STR of 3 × 10^8^ and 2 × 10^9^ cells·mL^−1^, respectively. C: inoculation was performed with a pellet obtained after centrifugation of 1,500 mL of pulp, corresponding to an initial biomass concentration in the 2 L-STR of 2 × 10^9^ cell·mL^−1^.

As seen on Figure 3A, experiments B and C began with a biomass concentration of 3 × 10^9^ cells·mL^−1^. However, in C, the inoculum was first centrifuged to reduce its volume. The experiment A began with a biomass concentration approximately 10 times lower than the others. pH varied only a little during experiments and remained approximately 1.50 (± 0.15). In contrast, redox potential quickly decreased to less than 700 mV vs. SHE when biomass concentration was higher than 10^9^ cells·mL^−1^ in the liquid phase (Figure 3B). The Eh decrease was much slower when biomass concentration was lower (experiment A). In this experiment, fresh inoculum was added after 14 days (by the addition of a pellet resulting from the centrifugation of 1.5 L of pulp from microbial growth on sulfur) to reach approximately 3 × 10^9^ cells·mL^−1^ as in experiments B and C. A linear decrease of the redox potential was observed.

Despite the enrichment with 1% CO_2_ of the injected N_2_ gas, no increase of the biomass in the liquid phase was noticed during B and C experiments. The concentration even decreased at the end of the experiment. In experiment A, an increase was noticed in the liquid phase during the 4 first days, but it then decreased back to the initial concentration, meaning that microbial growth did not occur or new biomass was attached to the solid.

The inoculum at 35°C used in this experiment was composed of *At. caldus* and *Sb. thermosulfidooxidans* species. The microbial community structure, determined by diversity fingerprints, had the same changes in the three experiments (data not shown). There was a switch from a bacterial community essentially composed of *At. caldus* after microbial growth on sulfur, to a community at the end of the experiments in which *Sulfobacillus* was largely dominant (91% in B and 95% in C) over *At. caldus* or even the only detected bacterium (in A).

**Figure 4:**
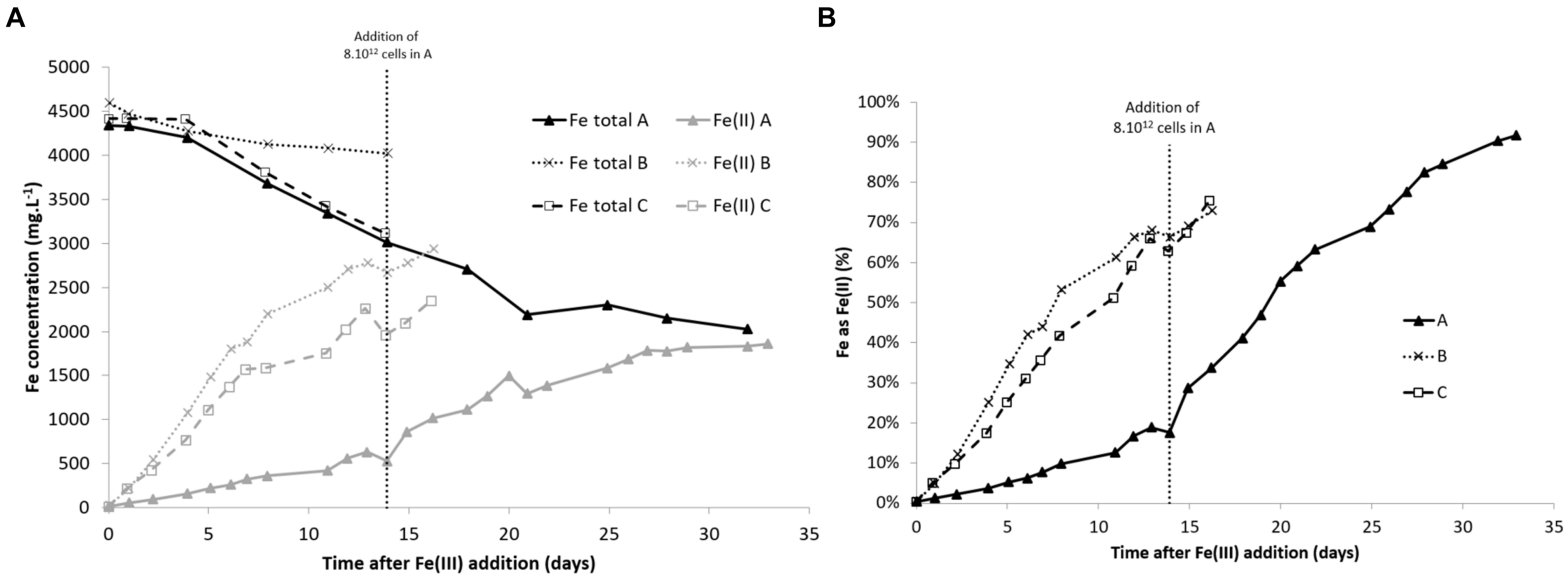
Total Fe and Fe(II) concentrations **(A)** and %Fe as Fe(II) **(B)** over time in bioreduction experiments with different initial biomass concentrations. Conditions A and B: inoculations were done with 200 mL and 1,500 mL, respectively, corresponding to an initial biomass concentration in the 2 L-STR of 3 × 10^8^ and 2 × 10^9^ cells·mL^−1^, respectively. C: inoculation was performed with a pellet obtained after centrifugation of 1,500 mL of pulp, corresponding to an initial biomass concentration in the 2 L-STR of 2 × 10^9^ cells·mL^−1^.

Fe(II) concentrations increased quickly after Fe(III) addition and then stabilized after 12 days (Figure 4). In the experiment with lower biomass concentration in the liquid phase, the production of Fe(II) was slower than in the other experiments, but the addition of cells after 14 days marginally increased the Fe(III) reduction rate. The total Fe concentrations decreased in most experiments. This may be caused by precipitation. Acidophilic bioleaching systems are often subject to precipitation due to high SO_4_^2−^ and Fe(III) concentrations, even though the pH stays lower than 2.0 (Mahmoud et al., 2017). However, the reasons that explain the difference of precipitation rates in the experiments are unclear.

Table 2 gives the mean and maximal rates that were measured during bioreduction experiments. Similar bioreduction rates were observed with another consortium cultivated at 46°C (see Supplementary material). A maximal bioreduction rate of 11.4 mg·L^−1^·h^−1^ and a mean value of 8.4 mg·L^−1^·h^−1^ were observed with this consortium, composed of *Sb. acidophilus* and traces of *At. caldus*, in the presence of 4 × 10^8^ cells·mL^−1^. However, it is not possible to directly compare both bioreactors, or to conclude on the improvement of bioreduction efficiency of one consortium compared to the other, because some differences occurred in the operating conditions (in particular, CO_2_ enrichment). Moreover, community composition monitoring revealed that *At. caldus* was a dominant bacterium at the end of the bioreduction phase at 46°C. This result is unexpected and cannot currently be explained.

**Table 2:** Maximal and mean bioreduction rates (in mg·L^−1^·h^−1^) obtained in bioreduction experiments.

Experiment	A	A after cell addition	B	C
Maximal rate	2.0	13.9	12.2	9.9
Mean rate	1.7	5.4	9.8	7.8

### 3.2 Continuous mode experiments

Operating processes in continuous mode rather than in batch mode generally makes it possible to improve reaction kinetics (Morin and d’Hugues, 2007). Furthermore, it has just been demonstrated that the aerobic growth phase on sulfur influences the Fe(II) bioreduction phase. The operation in continuous mode can facilitate the study of the influence of the change of operating conditions during the growth on bioreduction. A mini-pilot was therefore set up, and the influence of the composition of the growth medium on the bioreduction kinetics was determined at 35°C.

#### 3.2.1 Physico-chemical parameters

In the continuous mode, the influence of the biomass concentration was evaluated by modifying the growth medium composition of the inlet solution. During the first stage, ABS-TE was used with 100 μM of Fe(II). During the second stage, 0Km medium was used with 1.5 g·L^−1^ of Fe(II) added as ferrous iron sulfate. The main results are given in Table 3.

**Table 3:** Results obtained during the pilot operation of bioreduction.

		Stage 1	Stage 2
R1—Biomass growth	HRT (h)	73	63
	Biomass concentration (cells·mL^−1^)	2 × 10^9^	5 × 10^9^
R2—Bioreduction	HRT (h)	135	111
	Eh (mV vs. SHE)	680	650
	Biomass concentration (cells·mL^−1^)	2 × 10^9^	4 × 10^9^
	% of Fe as Fe(III)	53%	27%
	Fe(III) bioreduction (mg·L^−1^·h^−1^)	8.8	19.5

During the first stage (ABS-TE medium), redox potential slowly increased up to 680 mV vs. SHE in R2 due to the starting of the bioreactor from a low value (less than 650 mV vs. SHE). During the second stage (0Km medium), the redox potential decreased and stabilized approximately 650 mV vs. SHE. The total Fe concentration was stable, while Fe(III) concentration decreased to less than 1 g·L^−1^ during the second stage. The bioreduction rate in R2 was estimated to be approximately 9 mg·L^−1^·h^−1^ at the end of the first stage and increased up to 19.5 mg·L^−1^·h^−1^ during the second one. These values are higher than the values obtained in batch experiments.

#### 3.2.2 Microbial population dynamics

The biomass concentration in the liquid phase reached approximately 2 × 10^9^ cells·mL^−1^ in the sulfur oxidation R1 reactor during the first stage. During the second stage, the biomass concentration increased up to approximately 5 × 10^9^ cells·mL^−1^. The increase of nutrient concentrations sustained a larger biomass concentration in the liquid phase in R1. The same result was obtained in the bioreduction R2 reactor, in which the biomass increased from 2 × 10^9^ cells·mL^−1^ to 4 × 10^9^ cells·mL^−1^ in the second stage with more nutrients.

**Figure 5:**
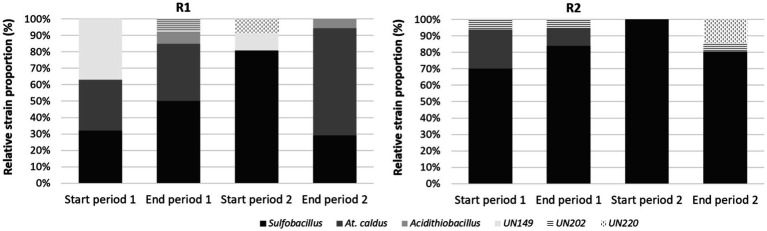
Diversity fingerprints in R1 and R2 reactors at the beginning and at the end of stages 1 and 2 in the continuous pilot operation.

The main bacteria detected on tRFLP fingerprints in the reactor R1 for microbial growth were *Sulfobacillus* species (corresponding either to *Sb. thermosulfidooxidans* or *thermotolerans* as both were retrieved by sequencing—see below) and *At. caldus* (Figure 5). Their relative abundance varied depending on the different stage. At the beginning of the first stage, they were quite equally distributed, with a third strain corresponding to an unidentified bacterium that could come from cross-inoculation since the continuous pilot was not operated in sterile conditions (32% *At. caldus*, 31% *Sulfobacillus,* and 37% unknown strain). *Sulfobacillus* proportion increased during stage 1 up to 50% and was even more dominant (81%) at the beginning of the second stage (9 days after the end of stage 1, i.e., more than 3 HRT). Then, at the end of the second stage, *At. caldus* was found as the main bacterium (65%). In the bioreduction reactor R2, the bacterial community was essentially composed of *Sulfobacillus* (more than 70%) and even exclusively at the beginning of the second stage. This is consistent with the batch experiments.

**Figure 6:**
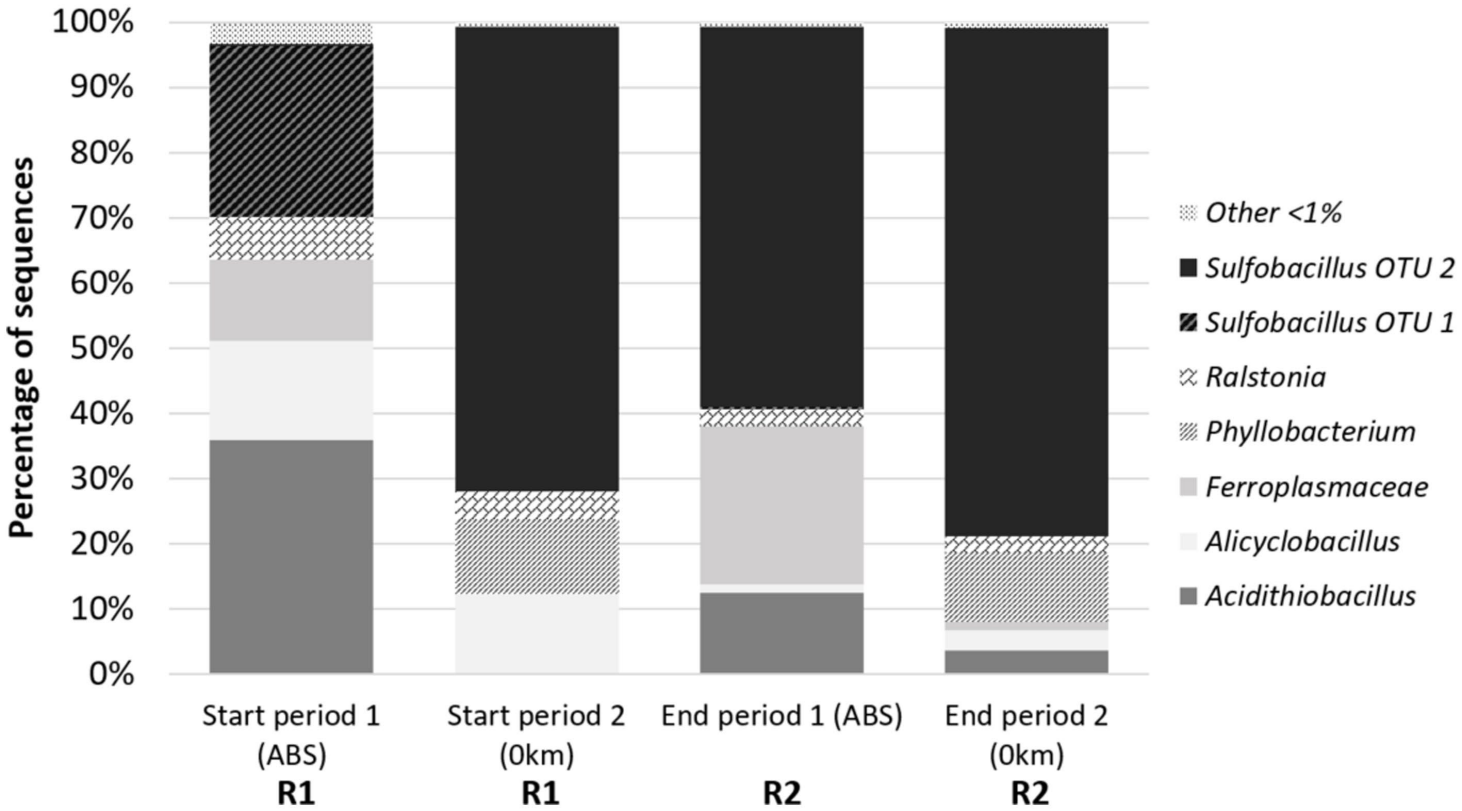
Taxonomic composition of the microbial communities in selected samples from R1 and R2 reactors of the continuous pilot operation.

To confirm and define more precisely the identity of the bacteria thriving in the continuous pilot, the taxonomic composition of the microbial community was determined by 16S rRNA gene metabarcoding on four samples selected from R1 (start of the stages 1 and 2) and R2 (end of the stages 1 and 2). The results confirmed the presence of the genera *Sulfobacillus* and *Acidithiobacillus* (Figure 6). Their occurrence within the whole community showed the same proportion tendency as the one revealed by tRFLP fingerprinting. They were both abundant (respectively, 26.4 and 35.8%) in R1 at the beginning of stage 1 on ABS medium, while *Sulfobacillus* dominated (71.3%) at the beginning of stage 2 on 0Km medium. *Sulfobacillus* was also dominant in R2 at the end of stage 1 (58.5%), and its proportion increased further at the end of stage 2 (78.0%).

In addition, metabarcoding allowed for a more detailed description of the community composition. At first, it revealed the occurrence of two OTUs affiliated to the genus *Sulfobacillus:* OTU-1 found only at the beginning of stage 1 in R1 and OTU-2 occurring at the start of stage 2 in R1 and at the end of stages 1 and 2 in R2. Species identification should be taken with precaution when considering a taxonomy based on partial 16S rRNA gene sequences (*ca.* 400 bp for the V3–V4 region). However, the comparison of OTU seed sequence with sequences of reference *Sulfobacillus* species clustered OTU-1 with *Sb. thermosulfidooxidans* and OTU-2 with *Sb. thermotolerans*. The *Acidithiobacillus* OTU seed sequence clustered with *At. caldus*-related sequences.

Other acidophilic genera were identified. In R1 for microbial growth, *Alicyclobacillus* was the third most abundant genus at both sampling times (15 and 12%). The theoretical *Hae*III tRF expected for *Alicyclobacillus*, determined by *in vitro* digestion of *Alicyclobacillus* 16S rRNA gene fragment used for tRFLP, matched with the unidentified signal UN149 retrieved on the tRFLP fingerprints. An OTU of the *Ferroplasmaceae* family was found in a proportion close to that of *Alicyclobacillus* but only at the start of stage 1 on ABS medium. It clustered with the genus *Ferroplasma* based on seed sequence comparison. In the bioreduction R2 reactor, the second more abundant bacterium after *Sulfobacillus* was the *Ferroplasmaceae* OTU (24.2%) at the end of stage 1. However, at the end of the second stage, the latter was in low proportion (1.3%), while *Sulfobacillus* was largely dominant (78.0%). *Alicyclobacillus* occurred only in low proportions in R2. In addition to these two well-known acidophilic genera, two others were identified, *Phyllobacterium* and *Ralstonia*. They most probably resulted from a contamination as they are soil and plant-associated bacteria unable to thrive in such extreme acidic conditions.

Among the acidophilic microorganisms, the members of the genera *Sulfobacillus*, *Ferroplasma,* and *Alicyclobacillus* have the ability to reduce Fe(III) and grow with Fe(III) reduction in anaerobic conditions (Malik and Hedrich, 2022), while the species *At. caldus* is not, contrary to other *Acidithiobacillus* species. During bioreduction at 35°C, *Sulfobacillus* was systematically the dominant genus found in batch and continuous modes. In the latter on ABS and 0Km media, 16S rRNA gene taxonomic analyses identified it more precisely as *Sb. thermotolerans*, which may have been cross-inoculated as the continuous reactor was not operated in sterile conditions. The genus *Ferroplasma* was also identified, however, only on ABS medium and in lower proportion. *Sulfobacillus* thus appeared as the main genus responsible for the bioreduction in our experiments.

#### 3.2.3 Nutrient consumptions

Nutrient concentrations (Table 4) were measured in each reactor to determine their consumption, which may come from the microbial growth and activity. In R2, it may also arise from the precipitation (as jarosite for example).

**Table 4:** Nutrient concentrations (in mg·L^−1^) in the STR during the stages of operation of the cascade pilot.

	Solution	K^+^	Mg^2+^	SO_4_^2−^	NH_4_^+^	PO_4_^3−^
Stage 1	Inlet	69.6	55.7	1,520	127	35.9
	Outlet of R1	5,959	47.2	12,707	59.3	18.3
	Outlet of R2	5,845	98.1	18,050	59.8	18.6
Stage 2	Inlet	273	52.3	6,402	979	435
	Outlet of R1	12,959	43.7	25,601	798	n.d.
	Outlet of R2	8,636	98.3	29,219	508	n.d.

K^+^ consumption could not be evaluated in R1 because large quantities of K_2_CO_3_ were added to maintain the pH, thus interfering with the nutrient consumption evaluation. However, K^+^ was consumed in R2 despite the fact that microbial growth was not detected. This consumption may arise from jarosite precipitation (which cannot be confirmed with SO_4_^2−^ concentrations due to the addition of Fe(III) sulfate) or from microbial consumption during bioreduction. The consumption of dissolved K^+^ reached 0.187 and 8.65 mmol·h^−1^ in R2 during stage 1 and 2, respectively. By comparison, a previous study from Hubau et al. (2023) reported a K^+^ microbial consumption of 0.083 mmol·h^−1^ during the acidophilic bioleaching of a polysulfidic mining residue in 0Km medium.

Mg^2+^ was slightly consumed in R1 during microbial growth (difference between inlet and outlet flow concentrations approximately 7 mg·L^−1^, i.e., 0.008 mmol·h^−1^). In R2, Mg^2+^ concentration increased, and this is still unexplained.

NH_4_^+^ consumption in R1 varied greatly from one stage of operation to another. The consumption (i.e., difference between inlet and outlet flow concentrations) reaches 68 mg·L^−1^ during stage 1 (0.104 mmol·h^−1^) and 181 mg·L^−1^ during stage 2 (0.319 mmol·h^−1^). The previous study from Hubau et al. (2023) reported a NH_4_^+^ consumption of the same order of magnitude (0.117 mmol·h^−1^) during the acidophilic bioleaching of a polysulfidic mining residue in 0Km medium.

Some authors propose the following reaction (Eq. 4) for cell synthesis with ammonium as a nitrogen source (Hatzikioseyian and Tsezos, 2006):


(4) $$\begin{array}{l} \frac{1}{5}CO_{2\left(g\right)}+\frac{1}{20}HCO_{3}^{-}+\frac{1}{20}NH_{4}^{+}+H^{+}+e^{-}\\ \to \frac{1}{20}C_{5}H_{7}O_{2}N+\frac{9}{20}H_{2}O \end{array}$$

Carbon dioxide uptake rate has been estimated to be close to 40 mg·L^−1^·h^−1^ by several authors in the case of the bioleaching of sulfidic minerals with acidophilic microorganisms (D’Hugues et al., 1997; Guezennec et al., 2018). Using this value and the stoichiometric ratio between C and N (5:1), the ammonium consumption linked to biomass growth and activity was estimated to be close to 0.455 mmol·h^−1^, which is very consistent with the values measured in R1.

NH_4_^+^ consumption in R2 was inexistent during the first stage and reached 290 mg·L^−1^ during the second stage (0.922 mmol·h^−1^). As the microbial growth was not detected in this reactor, it may arise from microbial activity of bioreduction or jarosite and ammoniojarosite precipitation. Similarly, PO_4_^3−^ was not consumed in R2 in the first stage.

**Figure 7:**
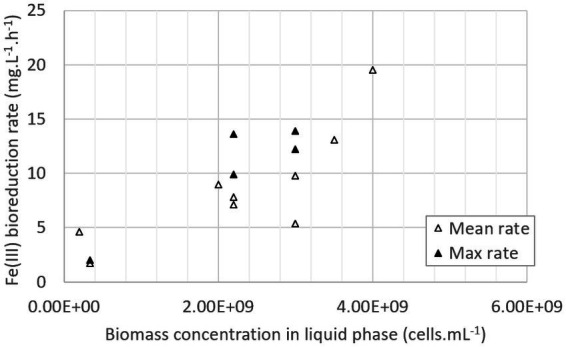
Fe(III) bioreduction rate as a function of the biomass concentration in the liquid phase (data from batch and continuous mode experiments).

To conclude, all the nutrients were not entirely consumed in the pilot, as if there were in excess whatever the growth medium composition. However, in the 0Km medium (stage 2), consumptions were higher than in the ABS-TE medium (stage 1), showing that larger nutrient concentrations favored their consumption (both from microbial growth and activity and from precipitation phenomena). The increase of the nutrient concentrations in the growth medium enabled to increase the microbial growth in the first step, which was clearly correlated with a higher Fe(III) bioreduction rate in the second step. The maximal value that was obtained was 19.5 mg·L^−1^·h^−1^. As shown in Figure 7, Fe(III) bioreduction rate is about to be proportional to the biomass concentration in the liquid phase. The correlation is given in Eq. 5, with v_Fe(III) bioreduction_ the bioreduction rate in mg·L^−1^·h^−1^ and X_liq_ the concentration of biomass in the liquid phase in cells·mL^−1^:


(5) $$v_{\mathrm{Fe}\left(\mathrm{III}\right)\mathrm{bioreduction}}=3.46\times 10^{-9}\;X_{liq}+1.55\;\mathrm{with}\;R^{2}=0.684$$

## 4 Discussion

Table 5 gathers the results of the different experiments. It showed that the presence of microorganisms highly increased the reduction rate compared to the abiotic experiment (0.13 mg·L^−1^·h^−1^). A mean bioreduction rate of 1.7 mg·L^−1^·h^−1^ was obtained in batch mode with 10^8^ cells·mL^−1^ in the liquid phase.

**Table 5:** Summary table of biomass concentration in the liquid phase (in cells·mL^−1^), maximal and mean bioreduction rates (in mg·L^−1^·h^−1^), and population dynamics in bioreduction experiments at 35°C in batch and continuous mode.

Conditions	ABS-TE batch	ABS-TE batch	ABS-TE batch	ABS-TE continuous	0Km continuous
Liquid phase biomass concentration during bioreduction (cells·mL^−1^)	3 × 10^8^	2 × 10^9^	2 × 10^9^	2 × 10^9^	5 × 10^9^
Maximal bioreduction rate (mg·L^−1^·h^−1^)	2.0	12.2	9.9	-	-
Mean bioreduction rate (mg·L^−1^·h^−1^)	1.7	9.8	7.8	8.8	19.5
Consortium composition	*At. caldus* and *Sulfobacillus*				
Composition after growth phase	*At. caldus*			50% *Sulfobacillus* and *At. caldus*, *Alicyclobacillus*	65% *At. caldus*, *Sulfobacillus*, *Alicyclobacillus*
Composition at the end of the experiment	*Sulfobacillus*	91% *Sulfobacillus, At. caldus*	95% *Sulfobacillus, At. caldus*	*Sb. thermotolerans*	*Sb. thermotolerans*

Bioreduction experiments highlighted an increase of the bioreduction rate with an increase of the initial biomass concentration in the liquid phase (up to 9.8 mg·L^−1^·h^−1^ of mean rate and 12.2 mg·L^−1^·h^−1^ of maximal rate when biomass concentration was approximately 10^9^ cells·mL^−1^ in the liquid phase, compared to 1.7 mg·L^−1^·h^−1^ in the presence of 10^8^ cells·mL^−1^). Similar results were observed in continuous mode. The increase of inorganic nutrient concentrations in 0Km medium enabled to detect a higher biomass concentration in the liquid phase than the ABS-TE medium, resulting in a higher Fe(III) bioreduction rate. A value of 20 mg·L^−1^·h^−1^ was observed in continuous mode and 0Km medium. The higher consumptions in 0Km medium compared to the ABS-TE medium showed that larger nutrient concentrations favored their consumption (both from microbial growth and activity and from precipitation phenomena). The availability of the nutrients may not be the only parameter that is at stake. In bioleaching systems, dissolved oxygen is known to be a key factor to obtain high performances. It was demonstrated that below a certain value of dissolved oxygen (approximately 2 mg/L), even though dissolved oxygen was available, the bioleaching kinetics were negatively affected (Morin and d'Hugues, 2007). A similar phenomenon may appear for the nutrients. This phenomenon may be due to mass transfer limitations or complexation.

The bioreduction kinetics obtained at 35°C in our study are in the same order of magnitude than the ones of Kucera et al. (2012) (26.1 mg·L^−1^·h^−1^), Pronk et al. (1992) (25.6 mg·L^−1^·h^−1^), and Breuker and Schippers (2024) (19.9 mg·L^−1^·h^−1^) but was measured in the presence of *Sb. thermotolerans* as the main species found after the bioreduction. This species previously demonstrated its ability to reduce Fe(III) (Malik and Hedrich, 2022), but there were, to our knowledge, no data on the kinetics obtained. It should be noticed that no microbial growth was observed in the liquid phase in all experiments at 35°C under anaerobic conditions, despite the fact that *Sulfobacillus* species were reported to grow under anaerobic conditions.

A linear correlation was observed between biomass concentration in the liquid phase and bioreduction rates, whether in batch or continuous mode. Even if a consequent proportion of microorganisms may be attached to sulfur particles, the determination of biomass concentration in the liquid phase by cell count is still a reliable indicator of the biomass available for Fe(III) bioreduction, both in batch and continuous mode. This result could also be improved by considering the biomass attached to sulfur particles, in particular in the case of higher sulfur content than in our study (1% or 2.4% (w/w)) which should increase the proportion of attached bacteria. This may be achieved by collecting solid particles and detaching bacteria before cell counts. The microbial DNA could also be extracted from planktonic bacteria and from the ones attached to the solids after their separation from the liquid phase, and gene abundance of total bacteria, determined by quantitative PCR, used as a marker of cell abundance. When optimized, these approaches have given comparable results to evaluate biomass of bacteria in batch bioleaching reactors (Hedrich et al., 2016).

*At. caldus* was detected in large quantities after sulfur oxidation. Since it is not known to reduce Fe(III), its presence might have provided organic carbons in the bioreduction reactor. Both *Sulfobacillus* and *Ferroplasma* genera detected in this study scavenge carbon from the excretion of chemolithotrophs or dead cell remains. This might also be the case for *Alicyclobacillus* which was found after sulfur oxidation but was a minor strain during bioreduction despite its ability to develop and reduce Fe(III). Like some other primary producers, *At. caldus* produces glyconic acid as an exudate, an organic compound that can be metabolized by *Sulfobacillus* species, including *Sb. thermosulfidooxidans*, as a specific metabolic trait that would give Sulfobacilli a competitive advantage in bioleaching by relation to other mixotrophs (Ñancucheo and Johnson, 2010; Justice et al., 2014).

Complementary molecular methods based on the widely used 16S rRNA gene marker have been applied to monitor community diversity dynamics in this study. They gave comparable results and evidenced *Sulfobacillus* strains as the iron reducers in bioreduction conditions. tRFLP fingerprinting has the advantage of being fast and easy to implement for a daily monitoring and is still acknowledged as powerful for profiling microbial community dynamics in relation to varying conditions in processes (De Vrieze et al., 2018). This is particularly true in bioleaching, because on the one hand such a selective environment leads to reduced microbial diversity and on the other hand the diversity of bioleaching strains is well documented. However, since only terminal restriction fragments are considered, a disadvantage is that two distinct sequences carried by different strains and sharing a terminal restriction site will not be distinguished. This happened here for both *Sulfobacillus* species present in the reactors and which have the same tRF. Metabarcoding of the 16S rRNA gene provided a more detailed description of the community composition and notably highlighted the presence of two species from the genus *Sulfobacillus*. This approach is more difficult to implement on a daily base but gives the needed knowledge on community diversity and the taxa to follow. By crossing the results, metabarcoding can be used to further improve tRFLP, e.g., by applying multiple restriction enzymes to better distinguish two species of a same genus, so that the main identified taxa can be detected and their evolution monitored. The two methods are complementary, and community monitoring in bioleaching will be improved if they are more generally combined.

## 5 Conclusion

Reductive bioleaching is one of the metallurgical options that are currently studied to extract Co from limonite. To design the bioprocess, Fe(III) bioreduction rates were determined in this study in the presence of different microbial consortia at different temperatures. In STR run in batch mode with a consortium cultivated at 35°C, a maximal bioreduction rate of 12.2 mg·L^−1^·h^−1^ and a mean value of 9.8 mg·L^−1^·h^−1^ were obtained. It was demonstrated that Fe(III) bioreduction rate was proportional to the biomass concentration in the liquid phase. Almost no evolution of the biomass in the liquid phase was noticed during bioreduction experiments. In continuous mode, the bioreduction rate was higher than the one measured in batch mode. It was estimated approximately 9 mg·L^−1^·h^−1^ with ABS-TE growth medium and increased up to 19.5 mg·L^−1^·h^−1^ with 0Km medium (richer medium). Even if nutrients were in excess in both growth media, the increase of nutrient concentrations sustained a larger biomass concentration in the liquid phase after sulfur oxidation. The microbial community structure switched from a bacterial community essentially composed of *At. caldus* after microbial growth on sulfur, to a community in which *Sulfobacillus* was largely dominant at the end of the bioreduction.

## Data availability statement

The datasets presented in this study can be found in online repositories. The names of the repository/repositories and accession number(s) can be found at: https://www.ncbi.nlm.nih.gov/genbank/, PRJEB71394.

## Author contributions

AH: Conceptualization, Investigation, Methodology, Supervision, Visualization, Writing – original draft. CJ: Methodology, Validation, Visualization, Writing – original draft. HT: Investigation, Writing – review & editing. DP-H: Investigation, Methodology, Validation, Writing – review & editing. CB: Investigation, Writing – review & editing. A-GG: Conceptualization, Project administration, Supervision, Validation, Writing – review & editing.

## Footnotes

^1^ https://blast.ncbi.nlm.nih.gov/Blast.cgi
