# Supplementary material for: Fe(III) bioreduction kinetics in anaerobic batch and continuous stirred tank reactors with acidophilic bacteria relevant for bioleaching of limonitic laterites
Source: Front Microbiol. 2024 Mar 11;15:1358788. doi: 10.3389/fmicb.2024.1358788 (PMC10964485; doi:10.3389/fmicb.2024.1358788)
Supplement: Supplementary file 1 [file Data_Sheet_1.docx]

Supplementary Material

Fe(III) bio-reduction kinetics in anaerobic batch and continuous stirred tank reactors with acidophilic bacteria relevant for bioleaching of limonitic laterites

Agathe Hubau*, Catherine Joulian, Hafida Tris, Douglas Pino-Herrera, Camille Becquet, Anne-Gwénaëlle Guezennec

*** Correspondence:** Agathe Hubau: a.hubau@brgm.fr

# Material and methods with the 46 °C consortium

The microbial consortium that was used in this study was provided by Prof. Barrie Johnson from Bangor Acidophile Research Team at Bangor University, UK. Upon reception, the consortium was subcultured in Acidophilic Basal Salts (ABS) medium (Ñancucheo et al., 2016) supplemented with 0.5%(w/v) elemental sulfur in shake flasks, at their defined temperature. *Sb. acidophilus* was the predominant species with traces of *At. caldus*.

Microbial growth was first carried out in aerobic mode on elemental sulfur at 46 °C in a 2 L-STR inoculated with 10%(v/v). Bioreactor was inoculated in ABS-TE medium supplemented with 1%(w/w) laboratory-grade sulfur and the air flow rate was 425 nmL.min^-1^. The stirring speed was set to 500 rpm. After 3 days, the biomass concentration in the liquid phase reached 5.10^8^ cell.mL^-1^ and the reactor was turned to anaerobic conditions by sparging nitrogen (425 mL.min^-1^). 4.8 g.L^-1^ Fe(III) was added as ferric iron sulfate.

In complement to microscopic cell counts, quantitative *real-time* PCR (qPCR) assays targeting the 16S rRNA gene were used to estimate bacterial biomass, using primers 341F and 534R and the procedure described by Hedrich et al. (2016). Real-time data and gene copies numbers were obtained with the CFX Manager software (BioRad), and results are expressed as gene copies per mL of pulp.


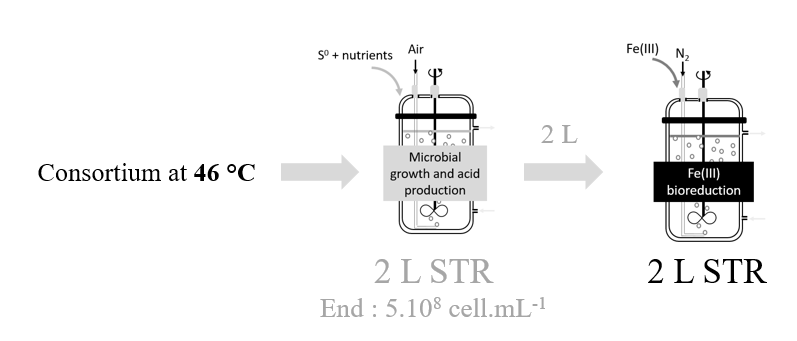


**Figure S1.** Schematic diagram of the experiments performed in batch mode at different initial biomass concentration at 46 °C.

# Results

The Fe(III) bioreduction ability was determined with the consortium at 46°C, mainly composed of *Sb. acidophilus*. Microbial growth was first performed aerobically. In three days, pH decreased to less than 1.2 (Figure S2) and the biomass concentration reached around 5.10^8^ cell.mL^-1^ (Figure S2).When the reactor was switched to bioreduction conditions, 4.5 g.L^-1^ Fe(III) was added. Redox potential started to decrease from 850 mV vs SHE. Figure S2 shows Fe(II) concentration over time: the increase of Fe(II) concentration corresponded to a maximal bioreduction rate of 11.4 mg.L^-1^.h^-1^ and a mean value of 8.4 mg.L^-1^.h^-1^ in the presence of 4.10^8^ cell.mL^-1^ (the same biomass concentration in the liquid phase than the experiments at 35 °C). However, it is not possible to directly compare both bioreactors, or to conclude on the improvement of bioreduction efficiency of one consortium compared to the other, because some differences occurred in the operating conditions (in particular, gas was supplemented with CO_2_ at 35 °C but not at 46 °C, which thus shortened the growth phase at 35 °C; the initial pH was at 1.5 at 35 °C compared to 1.7 at 46 °C).


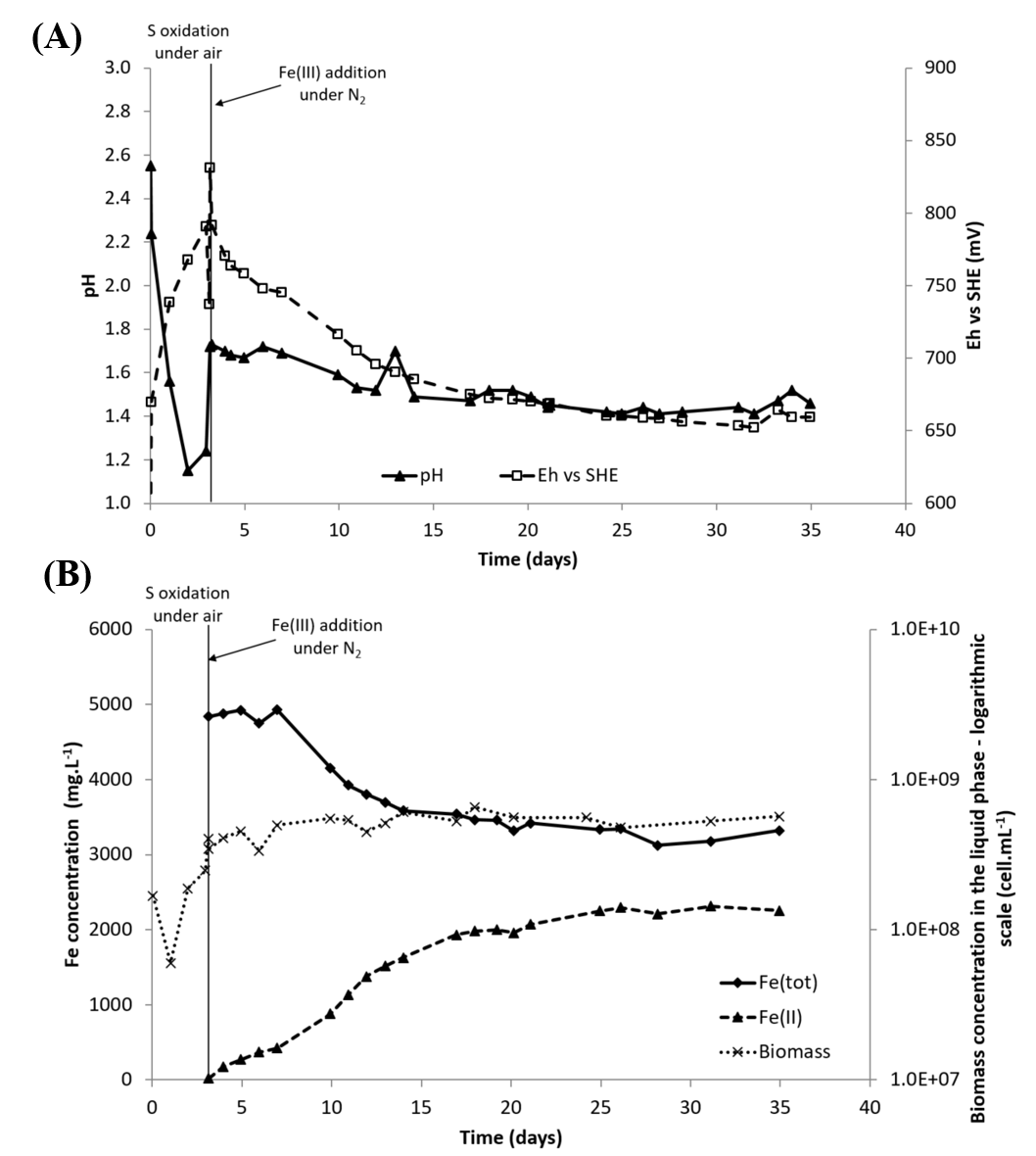


**Figure S2.** (A) pH and redox potential evolution and (B) total Fe and Fe(II) concentrations, and biomass concentration in the liquid phase in a two 2 L-STR for the determination of Fe(III) bioreduction rate at 46 °C.

Biomass in liquid phase did not increase in the presence of ferric iron sulfate (data not shown). However, the cell counting was not reliable, due to the presence of small particles of solids that interfere during counting. To overcome this bias, copy numbers of the 16S rRNA gene were used as a marker of biomass in the reactor, as they should be directly linked to the concentration of bacteria in a system (Figure S3). These measurements target biomass in the liquid phase and associated to solid particles, as microbial DNA were extracted from pulp samples. At the end of the growth phase (day 3), the concentration of gene copies reached 1.2x10^5^ copies/mL. At day 21, they were lower (0.9x10^4^ copies/mL). At the end of the experiments, concentration of gene copies had increased (1.4x10^7^ copies/mL). In this experiment, gene copies were much lower than cell counts and their evolution did not follow the same trend (data not shown). The concentration of DNA was close to the detection limit (Figure S3), showing a limited recovery of DNA from theses samples. Further research should be performed to obtain reliable indication of the biomass.


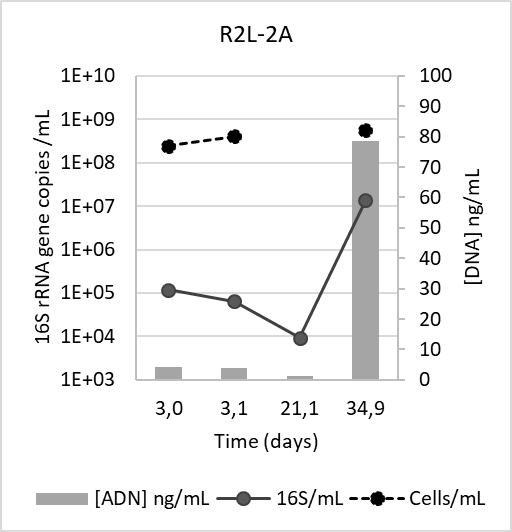


Figure S3. 16S rRNA gene copies and DNA concentration over time in the bioreactor with ferric iron sulfate at 46 °C.

Community compositions were monitored by tRFLP fingerprinting (data not shown) and revealed that *Sb. acidophilus* was the dominant bacterium (97%) and a low proportion of another tRF matching with tRF of *Sb. thermosulfidooxidans* or *Sb. thermotolerans*. At the end of the experiments, the community had shifted and *At. caldus* was a dominant bacterium while *Sulfobacillus* strains were in low proportion (less than 8%). Unidentified tRF were also found, UN202 representing 11% of the community. As explained, the UN149 tRF may correspond to *Alicyclobacillus* strains. As *At. caldus* is not known to reduce Fe(III) under anaerobic conditions, the unknown bacterium may be the main iron-reducer. The disappearance of *Sulfobacillus* is not clearly understood.
